# NPAS2 Compensates for Loss of CLOCK in Peripheral Circadian Oscillators

**DOI:** 10.1371/journal.pgen.1005882

**Published:** 2016-02-19

**Authors:** Dominic Landgraf, Lexie L. Wang, Tanja Diemer, David K. Welsh

**Affiliations:** 1 Veterans Affairs San Diego Healthcare System, San Diego, California, United States of America; 2 Department of Psychiatry & Center for Circadian Biology, University of California, San Diego, La Jolla, California, United States of America; Charité - Universitätsmedizin Berlin, GERMANY

## Abstract

Heterodimers of CLOCK and BMAL1 are the major transcriptional activators of the mammalian circadian clock. Because the paralog NPAS2 can substitute for CLOCK in the suprachiasmatic nucleus (SCN), the master circadian pacemaker, CLOCK-deficient mice maintain circadian rhythms in behavior and in tissues *in vivo*. However, when isolated from the SCN, CLOCK-deficient peripheral tissues are reportedly arrhythmic, suggesting a fundamental difference in circadian clock function between SCN and peripheral tissues. Surprisingly, however, using luminometry and single-cell bioluminescence imaging of PER2 expression, we now find that CLOCK-deficient dispersed SCN neurons and peripheral cells exhibit similarly stable, autonomous circadian rhythms *in vitro*. In CLOCK-deficient fibroblasts, knockdown of *Npas2* leads to arrhythmicity, suggesting that NPAS2 can compensate for loss of CLOCK in peripheral cells as well as in SCN. Our data overturn the notion of an SCN-specific role for NPAS2 in the molecular circadian clock, and instead indicate that, at the cellular level, the core loops of SCN neuron and peripheral cell circadian clocks are fundamentally similar.

## Author Summary

In mammals, circadian clocks are based on a core transcriptional–translational feedback loop. BMAL1 and CLOCK activate the transcription of *Per1-3* and *Cry1/2*. PER and CRY proteins inhibit BMAL1/CLOCK, and thus their own transcription. In *Clock*^*-/-*^ mice, NPAS2 can substitute for CLOCK in the suprachiasmatic nucleus (SCN), the major circadian pacemaker. However, peripheral tissues of *Clock*^*-/-*^ mice were reported to lack circadian rhythms. Since then, the protein CLOCK has been deemed essential for circadian rhythms in peripheral tissues. However, here we show that *Clock*^*-/-*^ peripheral cells and tissues exhibit stable, autonomous circadian rhythms. Furthermore, in *Clock*^*-/-*^ fibroblasts, knockdown of *Npas2* leads to arrhythmicity, suggesting that NPAS2 can compensate for the loss of CLOCK in peripheral cells as well as in SCN. Our data overturn the notion of an SCN-specific role for NPAS2, and instead indicate that the core loops of SCN neuron and peripheral cell circadian clocks are fundamentally similar. This finding redefines a basic principle of molecular circadian clock regulation in peripheral organs which are essential for many metabolic processes. Disturbances of these rhythms lead to disorders like diabetes, obesity, and cancer. Thus, understanding the molecular basis of peripheral circadian oscillators is essential to develop treatments against clock-related disorders.

## Introduction

Endogenous circadian (ca. 24 h) biological clocks have evolved in virtually all organisms in order to synchronize physiological processes and behavior with 24 h day/night cycles and to anticipate reliably recurring daily events. In mammals, the master circadian clock is located in the brain, in the hypothalamic suprachiasmatic nucleus (SCN) [1]. The SCN receives light information directly from the retina via the retino-hypothalamic tract, allowing it to synchronize to external light/dark cycles. Through direct and indirect signals, the SCN regulates subsidiary circadian clocks in peripheral tissues that coordinate rhythms of local physiological processes [2].

At the cellular level, the circadian clock is comprised of clock genes that interact in transcriptional-translational delayed negative feedback loops (TTLs) [3,4]. In the core TTL that is essential for rhythmicity, BMAL1 and CLOCK form heterodimers that activate the transcription of *Period* (*Per1*,*2*,*3*) and *Cryptochrome* (*Cry1*,*2*) genes. PER and CRY then form complexes that translocate into the nucleus and suppress the activity of BMAL1:CLOCK, thereby inhibiting their own transcription. When PER/CRY complexes are eventually degraded in a controlled fashion, this inhibition is relieved, BMAL1:CLOCK heterodimers become active again, and the ca. 24 h cycle begins anew.

The first known mammalian core clock gene was identified in a behavioral screen of chemically mutagenized mice, which yielded the *Clock-Δ19* mutant [5]. This mouse carries a mutation in intron 19 of the gene *Clock*, leading to expression of a non-functional CLOCK protein that competes with wild-type CLOCK for binding in molecular complexes. In constant darkness, heterozygous and homozygous *Clock Δ19* mice show long free-running periods of circadian locomotor activity rhythms, and homozygotes generally become arrhythmic, although this varies with genetic background. Consistent with the behavioral phenotype, molecular rhythm amplitudes in the SCN are severely reduced in homozygous *Clock-Δ19* mice, and peripheral tissues do not exhibit autonomous circadian oscillations [6–8]. These findings led to the initial assumption that CLOCK, like BMAL1, is an essential component of the TTL, such that circadian clocks are non-functional in the absence of CLOCK [9].

However, development of a mouse with a complete knockout of the *Clock* gene changed this concept. In contrast to *Clock-Δ19* mice, in constant darkness *Clock*^*-/-*^ mice show strong free-running rhythms that have a period ~20 min shorter than wild-type and do not become arrhythmic [10–12]. Although molecular rhythms in the SCN and liver of *Clock*^*-/-*^ mice are reduced in amplitude, they are stably expressed in animals kept in constant darkness. Conceivably, an unknown dimerization partner for BMAL1, which can substitute for CLOCK in *Clock*^*-/-*^ mice but unsuccessfully competes with the mutated CLOCK protein in *Clock-Δ19* mice, is capable of maintaining circadian rhythms in the total absence of CLOCK protein.

This unknown partner was identified as NPAS2, a paralog of CLOCK that dimerizes with BMAL1 to form transcriptionally active complexes [13]. Whereas knockout of the *Npas2* gene has no severe effects on behavioral rhythms in mice, double-knockout of *Npas2* and *Clock* leads to total arrhythmicity of behavior and of SCN clock gene expression. Interestingly, however, NPAS2 has been shown to have a role in controlling circadian rhythms only in the SCN, where NPAS2 can substitute for CLOCK, and in the forebrain, where NPAS2 is required for rhythmicity [13,14]. Even though NPAS2 is expressed in peripheral tissues and is in fact upregulated in livers of *Clock*^*-/-*^ mice [10], isolated peripheral tissues from *Clock*^*-/-*^ mice, no longer under the influence of the SCN, were reported to lack circadian rhythms [15]. Together, these results led to the current concept that NPAS2 can compensate for the loss of CLOCK in the SCN but not in peripheral tissues [9].

Here, we show that peripheral fibroblasts from *Clock*^*-/-*^; *mPer2*^*Luc*^ mice exhibit sustained, cell-autonomous circadian PER2 oscillations of similar quality to those of dispersed SCN neurons, which however were much less robust than those of SCN neurons in slice cultures, in which the reinforcing effects of SCN neuronal network interactions are largely preserved. Various peripheral tissues of *Clock*^*-/-*^ mice exhibited sustained, autonomous circadian rhythms, but with lower amplitude. Importantly, *Npas2* knockdown rendered *Clock*^*-/-*^ fibroblasts arrhythmic, demonstrating that NPAS2 can substitute for CLOCK to maintain rhythmicity in peripheral cells. Our data indicate that the role of NPAS2 in circadian clock function is similar in SCN and peripheral tissues.

## Results

### The SCN oscillator network attenuates effects of CLOCK deletion on the cellular circadian clock

**Fig 1:**
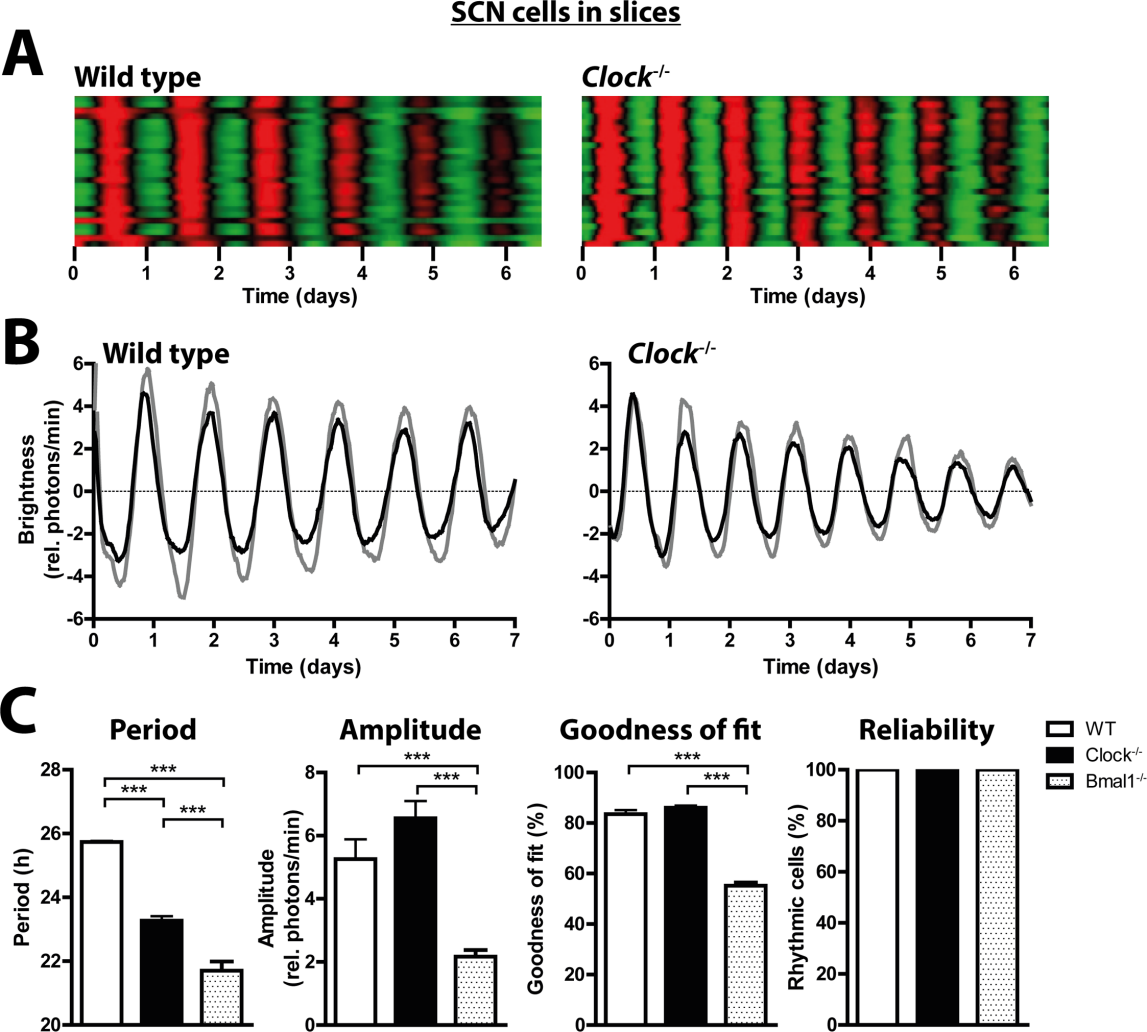
*Clock*^*-/-*^ SCN neurons show stable circadian rhythms in cultured SCN explants. (A) Raster plots of mPer2^Luc^ bioluminescence intensity of individual wild-type (left, n = 26) and *Clock*^*-/-*^ (right, n = 26) SCN cells in slices. Each horizontal line represents a single cell, with time in days plotted left to right. Values above and below the mean are shown in red and green, respectively. (B) mPer2^Luc^ bioluminescence rhythms of two representative rhythmic individual neurons (black and gray curves) in organotypic SCN slice cultures from wild-type mice (left) and *Clock*^*-/-*^ mice (right). (C) Mean circadian period, amplitude, and sine wave goodness-of-fit of cellular mPer2^Luc^ rhythms, and the percentage of rhythmic neurons in organotypic SCN slice cultures from wild type (white), *Clock*^*-/-*^ (black), and *Bmal1*^*-/-*^ (patterned) mice. *Bmal1*^*-/-*^ data are from Ko et al [18]. *Bmal1*^*-/-*^ SCN slices showed unusual lability of period, not reflected in these summary statistics. Data are shown as mean ± SEM; ***p≤0.001 (student’s t-test); or as percentage of cells that were significantly rhythmic; WT: n (rhythmic/total) = 75/75; *Clock*^*-/-*^: n = 79/79; *Bmal1*^*-/-*^: n = 80/80.

**Fig 2:**
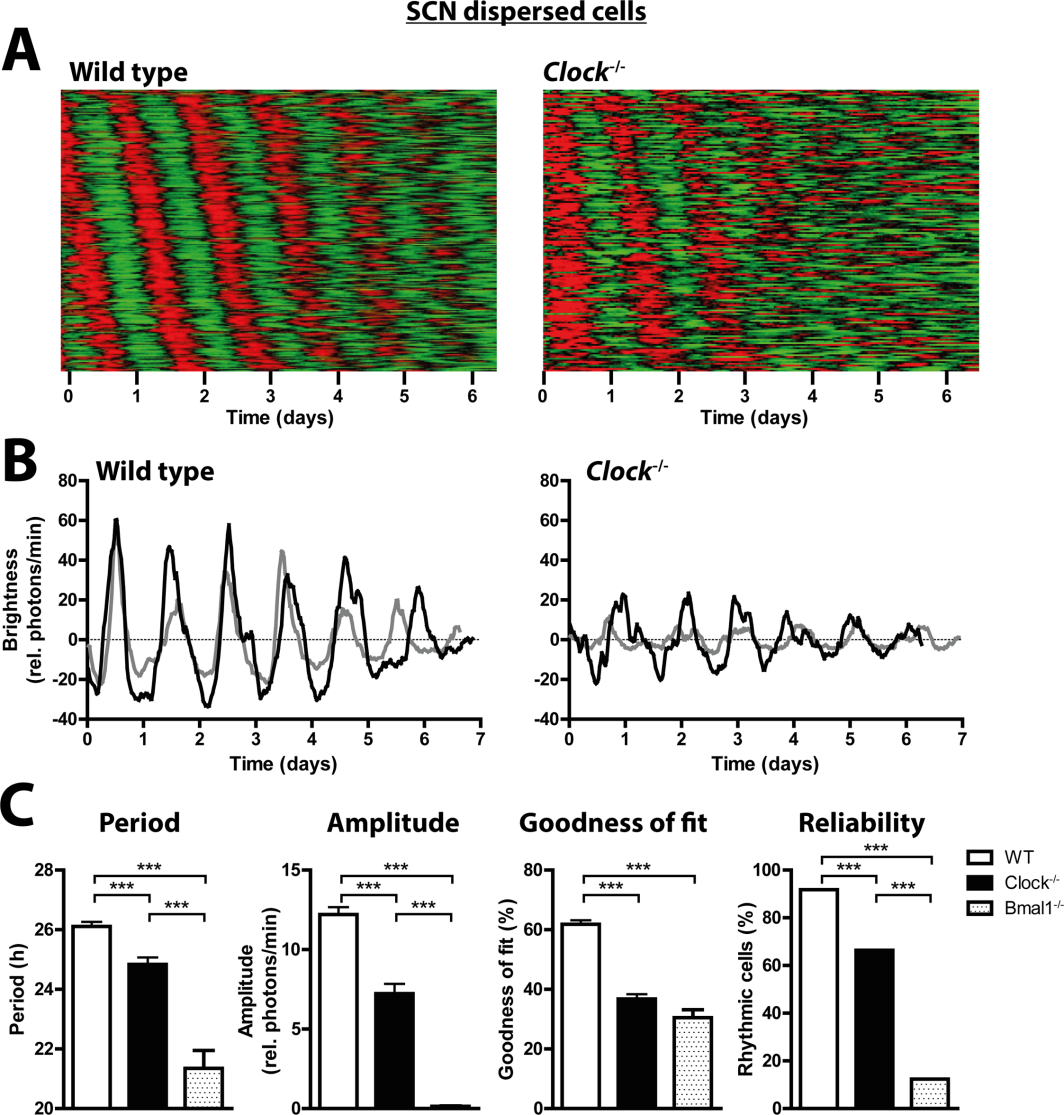
The SCN oscillator network is responsible for stable rhythms in *Clock*^*-/-*^ SCN neurons. (A) Raster plots of mPer2^Luc^ bioluminescence intensity of individual dispersed wild-type (left, n = 246) and *Clock*^*-/-*^ (right, n = 161) SCN cells. Data are presented as in Fig 1C. (B) mPer2^Luc^ bioluminescence rhythms of two representative rhythmic individual dispersed SCN neurons from wild type (left) and *Clock*^*-/-*^ mice (right). (C) Circadian period, amplitude, and sine wave goodness-of-fit of cellular mPer2^Luc^ rhythms, and the percentage of rhythmic neurons in dispersed SCN cultures from wild type (white), *Clock*^*-/-*^ (black), and *Bmal1*^*-/-*^ (patterned) mice. *Bmal1*^*-/-*^ data are from Ko et al [18]. Data are shown as mean ± SEM; ***p≤0.001 (student’s t-test); or as percentage of cells that are significantly rhythmic; WT: n (rhythmic/total) = 234/255; *Clock*^*-/-*^: n = 138/208; *Bmal1*^*-/-*^: n = 30/243.

*Clock*^*-/-*^ mice show relatively stable circadian rhythms in locomotor activity [10], an output of the circadian system that is directly controlled by the SCN. One possible explanation for this is that in SCN neurons NPAS2 can substitute for CLOCK in dimers with BMAL1, such that transcriptional activation at E-boxes and circadian oscillations are maintained [13]. Alternatively, specialized coupling among component cellular oscillators within the SCN neuronal network increases robustness against mutations of several clock genes [16,17], and this might also explain the preservation of locomotor activity rhythms in *Clock*^*-/-*^ mice. To investigate autonomous circadian clock function in SCN and peripheral cells and tissues of *Clock*^*-/-*^ mice, we crossed them with the mPer2^Luc^ reporter line and obtained *Clock*^*-/-*^ mice bearing the bioluminescent PER2 reporter. Similar to results previously reported for whole *Clock*^*-/-*^ SCN explants [13], single cells of cultured organotypic SCN slices of *Clock*^*-/-*^ mice displayed very stable circadian rhythms that were almost indistinguishable from those of wild-type SCN cultures (Figs 1A and 1B and S1A and S1 Dataset). Consistent with the behavioral phenotype, circadian periods of SCN cells of organotypic cultures from *Clock*^*-/-*^ mice were shorter than for wild-type mice. However, rhythm amplitude, goodness of fit, and number of rhythmic cells were unaffected by the mutation (Fig 1C). In contrast, when SCN cells were dissociated and studied in dispersed cultures lacking the specialized neuronal network that maintains synchrony of cells in SCN slices, the quality of their rhythms was reduced drastically (Figs 2A and 2B and S1B and S2 Dataset). Dispersed SCN cells from *Clock*^*-/-*^ mice exhibited not only shorter and less consistent circadian periods compared to wild-type cells, but also significantly reduced amplitudes, reduced goodness of fit, and a tendency toward smaller number of rhythmic cells (Fig 2C). For comparison, we re-analyzed data obtained previously from *Bmal1*^*-/-*^ SCN explants and dispersed SCN cells [18] using the same criteria used here for wild-type and *Clock*^*-/-*^ cultures. As previously reported [18], the SCN neuronal network partly compensated for the loss of BMAL1 (Fig 1C), whereas dispersed *Bmal1*^*-/-*^ SCN cells were mainly arrhythmic, and the few rhythmic dispersed *Bmal1*^*-/-*^ SCN cells had extremely low amplitudes (Fig 2C). Compared to *Bmal1*^*-/-*^ SCN cells, *Clock*^*-/-*^ SCN cells showed much stronger rhythms (Figs 1C and 2C).

### Clock^-/-^ fibroblasts show circadian properties similar to those of dispersed Clock^-/-^ SCN neurons

**Fig 3:**
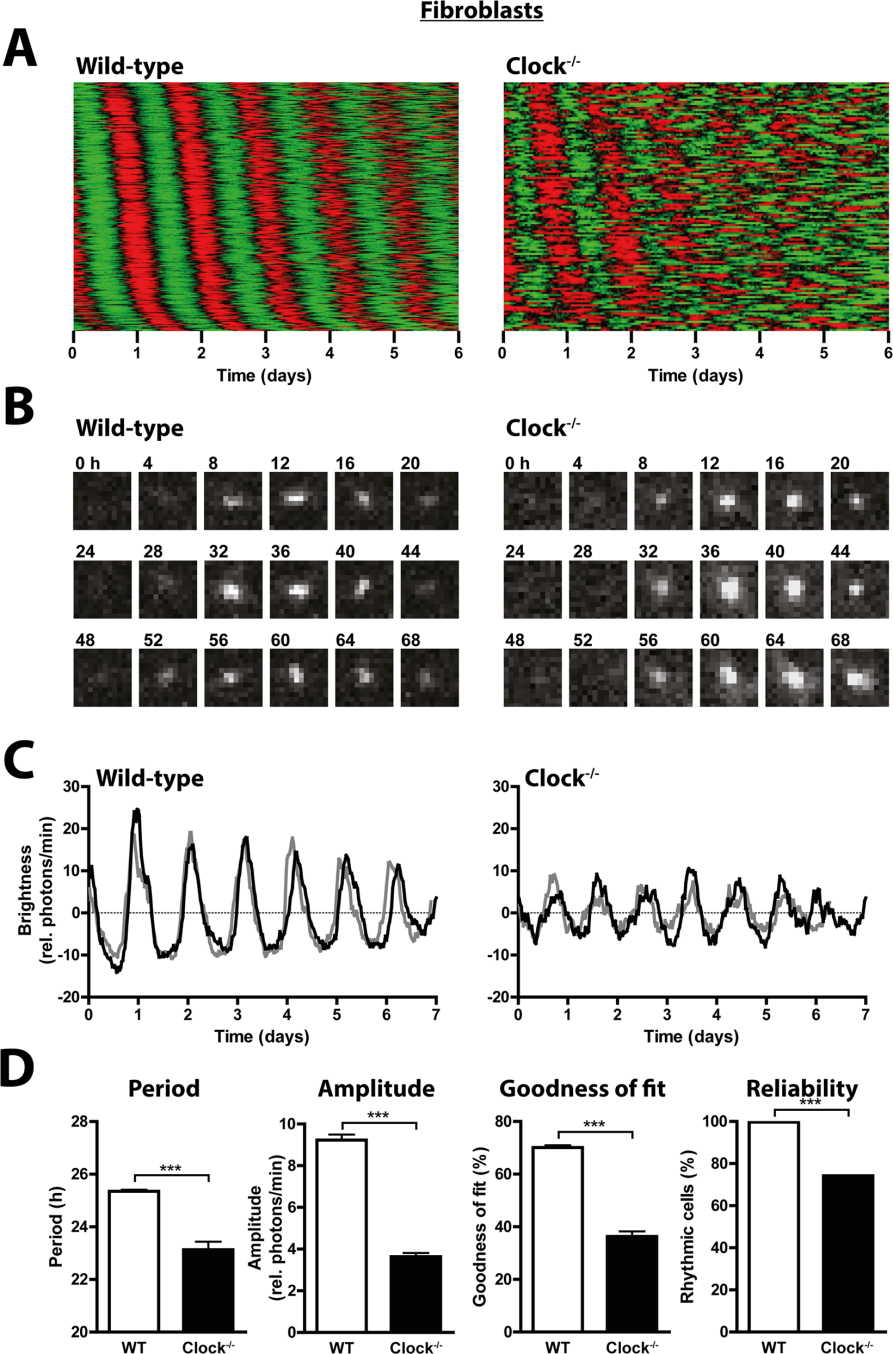
Dispersed *Clock*^*-/-*^ fibroblasts show circadian rhythms comparable to those of dispersed *Clock*^*-/-*^ SCN neurons. (A) Raster plots of bioluminescence intensity of individual dispersed wild-type (left, n = 320) and *Clock*^*-/-*^ (right, n = 121) SCN cells. Data are presented as in Fig 1C. (B) Images of mPer2^Luc^ expression of representative rhythmic wild-type (left) and *Clock*^*-/-*^ (right) fibroblasts. (C) Two representative mPer2^Luc^ bioluminescence rhythms of individual rhythmic wild-type (left) and *Clock*^*-/-*^ (right) fibroblasts. (D) Circadian period, amplitude, and goodness of fit of mPer2^Luc^ rhythms, and % of cells that were significantly rhythmic, for individual wild type (left) and *Clock*^*-/-*^ (right) fibroblasts. Data are shown as mean ± SEM; *p≤0.05, ***p≤0.001 (Student’s t-test); or % of cells rhythmic; ***p≤0.001 (Fisher’s exact test); WT: n (rhythmic/total) = 320/321; *Clock*^*-/-*^: n = 121/163.

Liver and lung tissues of *Clock*^*-/-*^ mice were previously shown to fail to exhibit circadian rhythms when isolated from the SCN [15]. To examine circadian clock function of individual peripheral cells from *Clock*^*-/-*^ mice, we assessed mPer2^Luc^ expression of primary fibroblasts in dissociated culture. Unexpectedly, *Clock*^*-/-*^ fibroblasts displayed relatively stable circadian rhythms (Figs 3A and 3B and S1C and S3 Dataset). As for dispersed SCN neurons, *Clock*^*-/-*^ fibroblasts exhibited shorter and less consistent circadian periods (Fig 3C). Furthermore, rhythm amplitudes and goodness of fit were significantly reduced compared to wild-type fibroblasts, and fewer cells were rhythmic.

### Peripheral Clock^-/-^ tissues exhibit circadian rhythms in the absence of SCN signaling

**Fig 4:**
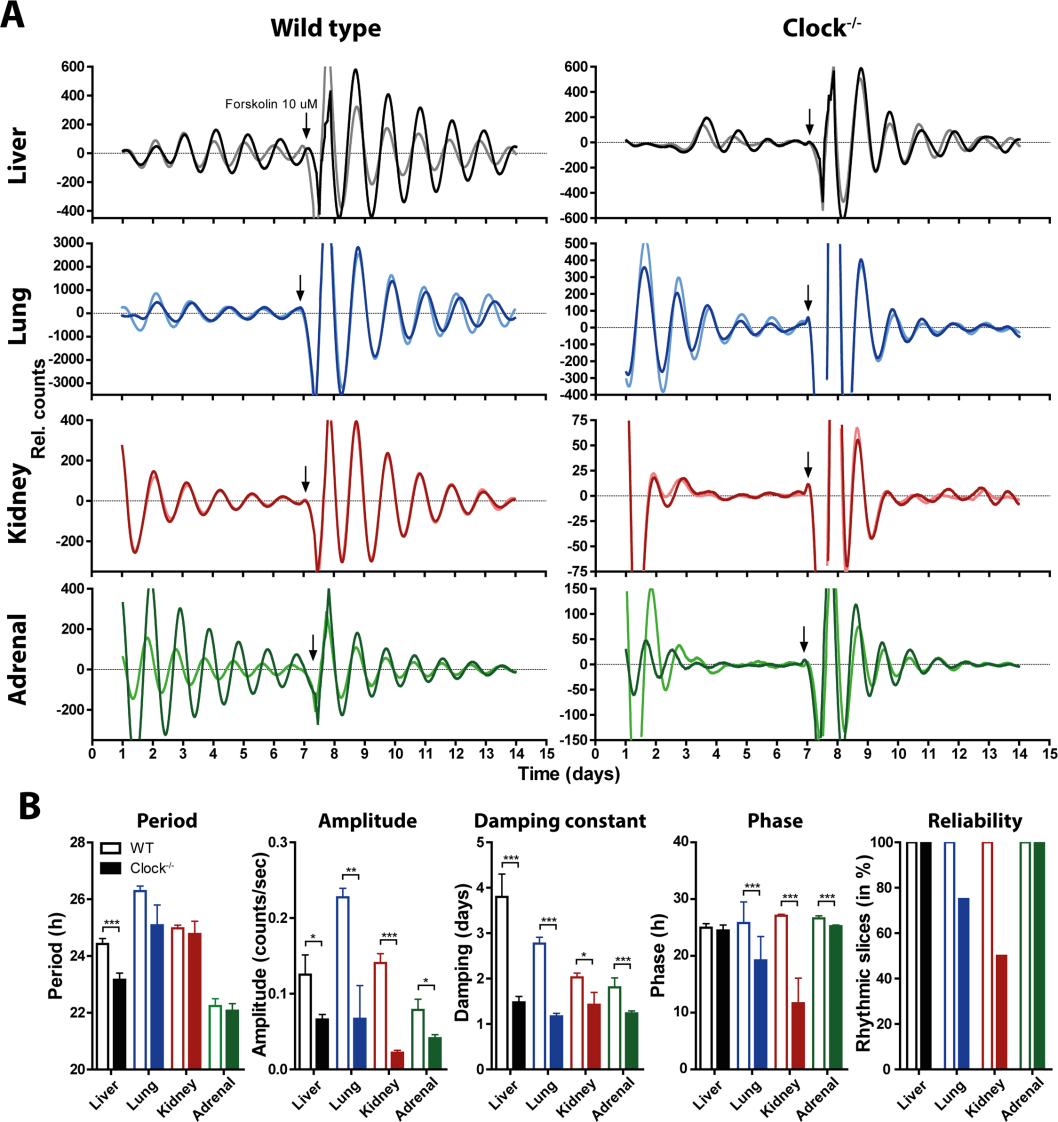
Peripheral organs of *Clock*^*-/-*^ mice can exhibit circadian rhythms *in vitro*. (A) Two representative mPer2^Luc^ bioluminescence rhythms of rhythmic organotypic liver (black), lung (blue), kidney (red), and adrenal (green) slice cultures from wild type (left) and *Clock*^*-/-*^ (right) mice. After ~7 culture days, samples were treated with 10 μM forskolin (arrow). Y-axis scales are adjusted to amplitudes for better visualization of data. (B) Circadian mPer2^Luc^ rhythm period, amplitude, damping constant (days to reach 1/e of initial amplitude), and phase of first peak after forskolin treatment, and % of slices from wild type (unfilled bars) and *Clock*^*-/-*^ mice (filled bars) that were significantly rhythmic after forskolin treatment (culture days 8–14). Data are shown as mean ± SEM; *p≤0.05, **p≤0.01, ***p≤0.001 (student’s t-test); or % of slices rhythmic; n = 8.

We cultured organotypic tissue slices of liver, lung, kidney, and adrenal from *Clock*^*-/-*^ mice and monitored tissue-autonomous mPer2^Luc^ rhythms at baseline and after administration of 10 μM forskolin to the culture medium. Surprisingly, all four tissues exhibited distinct circadian rhythms at baseline, and oscillations became more pronounced after treatment with forskolin Figs (4A and S2 and S4 Dataset). However, there were differences in quality of circadian rhythms between wild-type and *Clock*^*-/-*^ tissues and also among different tissues of the same genotype. At baseline, only *Clock*^*-/-*^ lung and kidney showed the short circadian period characteristic of *Clock*^*-/-*^ mice, whereas *Clock*^*-/-*^ liver and adrenal showed periods comparable to those of wild-type tissues (S3 Fig). However, after the forskolin treatment, only *Clock*^*-/-*^ liver exhibited shorter periods (Fig 4B). In all *Clock*^*-/-*^ tissues, rhythm amplitude was significantly lower than in wild-type tissues (Figs 4B and S3). In addition, damping of rhythms of *Clock*^*-/-*^ tissues, likely reflecting progressive desynchrony among cells, was faster than in wild-type tissues (Figs 4B and S3).

*Clock*^*-/-*^ and wild type tissues also differed in phase of circadian rhythms before and after resetting with forskolin (Figs 4 and S3).

Although we found rhythms in all four peripheral *Clock*^*-/-*^ tissues, not all individual explants showed significant rhythms. Without further synchronization, only 50% of *Clock*^*-/-*^ liver explants and 25% of *Clock*^*-/-*^ kidney explants were rhythmic, whereas all wild-type tissue explants and almost all *Clock*^*-/-*^ lung and adrenal explants showed significant rhythms (S3 Fig). Forskolin was able to induce rhythms in slices that had been arrhythmic before the treatment. After administration of forskolin, all *Clock*^*-/-*^ liver and adrenal explants, 75% of *Clock*^*-/-*^ lung explants, and 50% of *Clock*^*-/-*^ kidney explants were rhythmic (Fig 4B).

Although rhythm quality and proportion of rhythmic explants was lower in *Clock*^*-/-*^ tissues than in wild-type tissues, our findings reveal that peripheral tissues of *Clock*^*-/-*^ mice are clearly capable of generating self-sustained circadian rhythms in the absence of any SCN input signal.

### Peripheral Clock^-/-^ tissues exhibit circadian rhythms with altered phases in vivo

To test potential changes of circadian clock gene expression patterns *in vivo*, we collected liver, lung, kidney, and adrenal tissues from wild-type and *Clock*^*-/-*^ mice at 6 different time points throughout the day and examined mRNA levels of *Npas2*, *Bmal1*, *Per2* and various clock controlled genes with qPCR. In *Clock*^*-/-*^ livers and lungs, all genes showed significant circadian oscillations (S4 Fig and S1 Table). However, in *Clock*^*-/-*^ kidneys only *Bmal1* expression was significantly rhythmic, and in *Clock*^*-/-*^ adrenals all core clock genes, but not the clock controlled gene *Star* showed significant rhythms. Mean *Npas2* expression was drastically increased in *Clock*^*-/-*^ livers (S4 Fig and S1 Table). Similar but less pronounced effects on *Npas2* expression were seen in *Clock*^*-/-*^ lungs and adrenals. Interestingly, although all mice were entrained to the same LD cycle, almost all investigated genes of *Clock*^*-/-*^ tissues showed phase differences relative to wild-type tissues (S4 Fig and S1 Table).

### NPAS2 can compensate for the loss of CLOCK in peripheral cells

**Fig 5:**
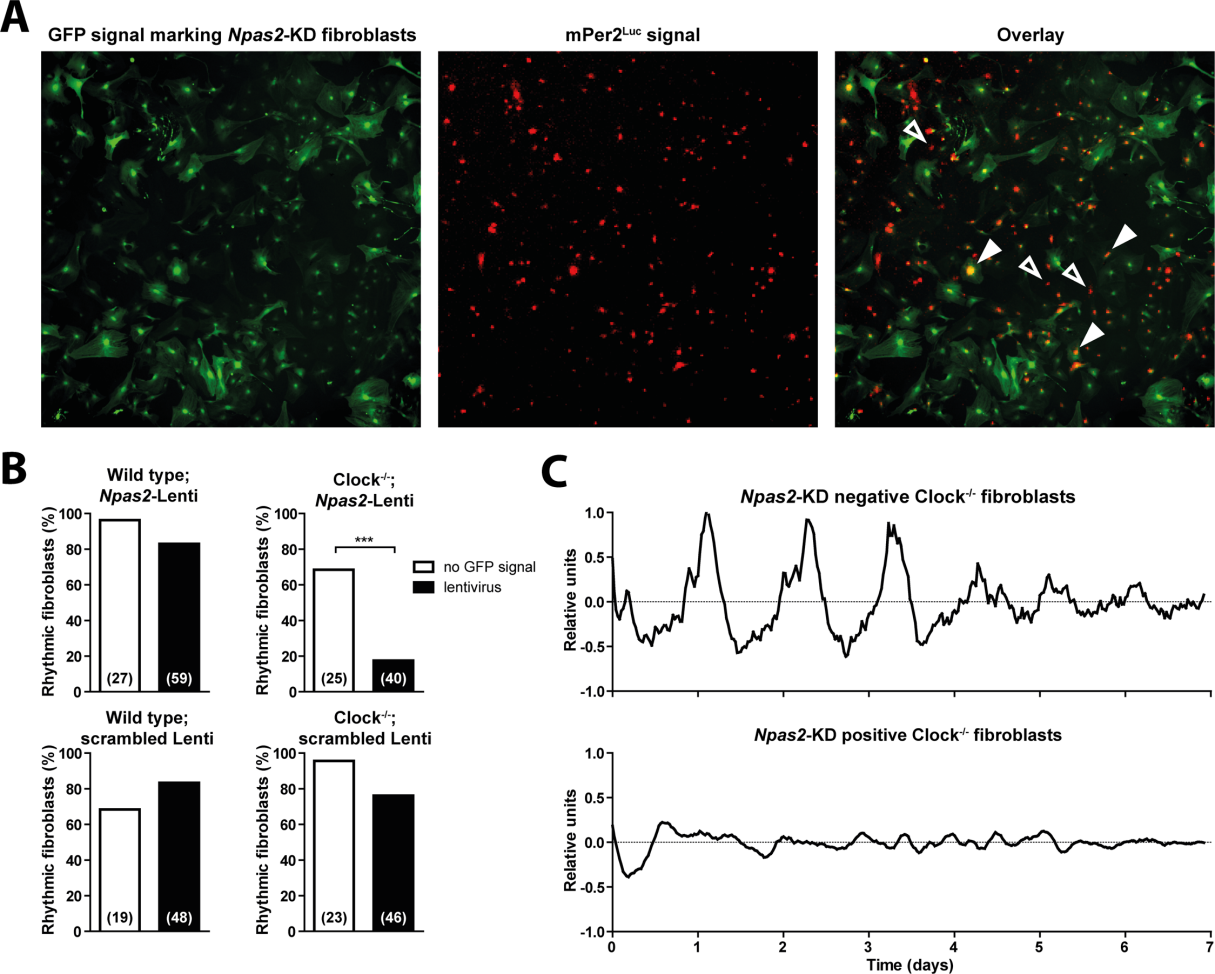
Knockdown of *Npas2* expression suppresses circadian rhythms in *Clock*^*-/-*^ fibroblasts. (A) Fibroblasts dispersed from wild type and *Clock*^*-/-*^ mice were treated with lentiviral vectors carrying an *Npas2*-KD or scrambled DNA sequence, as well as a GFP reporter. Simultaneous fluorescence and bioluminescence images of a representative field show GFP expression marking transfected cells (left, green) and mPer2^Luc^ bioluminescence from both transfected and untransfected cells (middle, red). The overlay shows that circadian rhythms could be measured from both transfected (filled arrowheads) and untransfected (unfilled arrowheads) fibroblasts in the same culture dish. (B) Percentage of wild type (left) and *Clock*^*-/-*^ (right) fibroblasts treated with *Npas2*-KD lentiviruses (top) or scrambled lentiviruses (bottom) that were significantly rhythmic. ***p≤0.001 (Fisher’s exact test); number of cells are given in parentheses. (C) mPer2^Luc^ rhythms of representative individual dispersed untransfected (top) and transfected (bottom) *Clock*^*-/-*^ fibroblasts from the same culture dish.

In the SCN and for rhythms of locomotor activity, the CLOCK paralog NPAS2 can compensate for the absence of CLOCK in *Clock*^*-/-*^ mice [13]. Since peripheral *Clock*^*-/-*^ cells and tissues also exhibit autonomous circadian rhythms, we asked whether NPAS2 plays a similar role in peripheral cells. We cultured primary wild-type and *Clock*^*-/-*^ fibroblasts and used lentiviral vectors expressing shRNAs to stably suppress *Npas2* expression. The average efficiency of the *Npas2*-knockdown was 60% (S5 Fig). Importantly, not all cells within a single culture dish were transduced by the viruses, allowing comparison of rhythms from affected and unaffected cells in the same dish, from the same mouse (Fig 5A). Most *Clock*^*-/-*^ fibroblasts with suppressed *Npas2* expression were arrhythmic, whereas most fibroblasts from the same *Clock*^*-/-*^ animals were rhythmic when *Npas2* expression was not affected (Fig 5B and 5C and S5 Dataset). In wild-type (*Clock*^*+/+*^) fibroblasts, knockdown of *Npas2* had no effect on the proportion of rhythmic cells, and infection with control viruses carrying a scrambled shRNA sequence did not change the proportion of rhythmic cells in either wild-type or *Clock*^*-/-*^ fibroblasts (Fig 5B). These findings suggest that NPAS2 is able to compensate for the loss of CLOCK by rescuing circadian rhythmicity in peripheral cells as well as the SCN.

## Discussion

Previous work developing and characterizing *Clock*^*-/-*^mice demonstrated that CLOCK is not essential for circadian oscillations of SCN clock gene expression or behavioral rhythms [10]. NPAS2 was identified as a substitute for CLOCK [13], rescuing circadian rhythmicity in its absence, but only in the SCN central clock and not in the periphery, where tissues failed to produce autonomous rhythms in the absence of CLOCK (and absence of SCN signals) *in vitro* [15]. In contrast, our data reveal that peripheral tissues and single fibroblasts of *Clock*^*-/-*^ mice are, in fact, capable of generating stable, autonomous circadian rhythms *in vitro*, albeit with diminished amplitude, reliability, and resetting capacity.

Our experiments indicate that when cultured in the absence of SCN input signals, liver, lung, kidney, and adrenal explants from *Clock*^*-/-*^ mice can exhibit stable circadian mPer2^Luc^ oscillations. However, the period and phase of these rhythms are different from wild-type controls, and these genotypic differences vary across tissues. *Clock*^*-/-*^ mice display a shorter free-running period of locomotor activity rhythms in constant darkness [10]; in our experiments, a corresponding shortening of circadian period is observed in SCN and in some (but not all) peripheral tissues cultured from *Clock*^*-/-*^ mice. Also, *Clock*^*-/-*^ mice show advanced phases of activity rhythms and enhanced phase shifting responses to light [11]. In cultured tissues and *in vivo*, the absence of CLOCK also affects phases of rhythmic expression of other core clock genes in a tissue-specific manner, suggesting a tissue-specific role for CLOCK in phase-setting and/or tissue-specific compensation mechanisms for the loss of CLOCK.

Compared to wild-type tissues, rhythms of *Clock*^*-/-*^ tissues generally exhibit lower amplitude, damp faster, and are less reliable. For example, when tested without prior synchronization by forskolin, only about 50% of *Clock*^*-/-*^ liver explants are rhythmic, and even when rhythms are present, they are of lower amplitude and damp faster than rhythms in wild-type liver explants. This may explain why rhythms in *Clock*^*-/-*^ peripheral tissues were not detected in a previous study by DeBruyne et al., although they observed low-amplitude rhythmicity in raw data of lung mPer2^Luc^ expression [15]. Thus, it is possible that different analysis methods or criteria for determining arrhythmicity explain the discrepancy between the studies.

Technical differences may also contribute to the different results. For example, DeBruyne et al. used mice that were backcrossed to the C57BL/6J strain for 8–10 generations and were *mPer2*^*Luc*^ heterozygous, whereas our mice were on a mixed background of C57BL/6J and Sv/129, and *mPer2*^*Luc*^ homozygous [15]. Finally, slight differences in culture procedures or media composition might differentially synchronize or amplify rhythms *in vitro*. Even simple changes of culture medium, for example, can temporarily synchronize cellular circadian oscillators, thereby revealing rhythmicity in an otherwise asynchronous population of peripheral cells [19]. In any case, whereas technical considerations could obscure rhythmicity, they cannot create rhythmicity de novo, so it seems safe to conclude that *Clock*^*-/-*^ peripheral cells and tissues are, in fact, capable of autonomous circadian oscillation.

Single-cell fibroblast experiments suggest that the weaker rhythms in *Clock*^*-/-*^ tissues are due to both lower single-cell amplitude and less consistent circadian period. However, some tissues compensate better for the lack of CLOCK than others. In the SCN, the neuronal network preserved in SCN slices compensates very effectively for the loss of CLOCK (Fig 1), as it does for other clock gene defects [16,17]. In the periphery, *Npas2* mRNA expression is more strongly upregulated in liver and adrenal than in lung or kidney, and this may account for the better rhythm amplitude and reliability in those *Clock*^*-/-*^ tissues relative to corresponding wild-type tissues (S4 Fig). The *Clock*^*-/-*^ kidney, which shows the least compensatory upregulation of *Npas2* expression of any of the four peripheral tissues studied, also shows the least reliable mPer2^Luc^ expression among explants *in vitro* (Figs 4 and S3), and the least consistent rhythms of gene expression *in vivo* (S4 Fig). However, even strong upregulation of *Npas2* in the liver does not fully substitute for CLOCK. The loss of CLOCK also leads to a decrease of BMAL1 [10], which is the binding partner of NPAS2. Thus, without sufficient levels of the binding partner BMAL1, even high amounts of NPAS2 may not be able to compensate completely for the loss of CLOCK.

Although intrinsic differences of molecular clock mechanism may exist across cell and tissue types, e.g. level of compensatory upregulation of *Npas2* expression, and these differences may contribute to the differing effects of *Clock* knockout that we observed across peripheral tissues, we found no evidence for such differences between SCN neurons and fibroblasts. In fact, effects of *Clock* knockout on cellular rhythmicity are remarkably similar in dispersed SCN neurons and fibroblasts. In both *Clock*^*-/-*^ and wild-type cells, rhythm amplitude is greater in SCN neurons than in fibroblasts. However, in both types of cells, amplitude, goodness of fit, and the proportion of rhythmic cells are all similarly reduced in the absence of CLOCK (Figs 2C and 3C). Consistent with this, *Clock*^*-/-*^ mice appear to accumulate BMAL1 in only ~10% of SCN neurons, suggesting a lower proportion of competent circadian oscillators in the SCN of these mice [10]. Together, this demonstrates that the previously observed robustness of SCN slice rhythms (and behavioral rhythms) to *Clock* knockout [10,13] can be explained by the specialized coupling within the SCN neuronal network that is lacking in peripheral tissues [16,17].

Earlier studies by DeBruyne et al. of tissues from double knockout mice deficient in both CLOCK and NPAS2 indicated that NPAS2 can substitute for CLOCK to maintain circadian rhythmicity in SCN but not in peripheral tissues [15]. However, PER2 rhythms in forebrain are abolished in NPAS2-deficient mice [14], implying an important role for NPAS2 in non-SCN clocks. Other studies have also suggested a role for NPAS2 in peripheral circadian clocks, although rhythms were not investigated in those studies [20–22]. NPAS2 binds to BMAL1 in whole brain and in peripheral tissues [10,21] and to E-boxes in the mouse liver [3], suggesting that it could be important for rhythmic transcription in peripheral cells. Importantly, we show here that knockdown of NPAS2 in *Clock*^*-/-*^ fibroblasts leads to complete arrhythmicity in most cells, thus establishing that NPAS2 can compensate for loss of CLOCK in peripheral cells as well as in SCN.

In summary, our results demonstrate that CLOCK is not essential for circadian clock function in peripheral tissues, because NPAS2 can substitute for CLOCK in these tissues, as it can in the SCN. However, this genetic compensation is incomplete, resulting in weaker rhythms in peripheral tissues of *Clock*^*-/-*^ mice. Neuronal network interactions specific to SCN further compensate for absence of CLOCK (as previously observed for other clock gene defects [16,17]), resulting in nearly normal circadian rhythms in SCN slices *in vitro* or locomotor activity *in vivo* [10,11,13]. Rhythmicity of gene and protein expression in peripheral tissues has previously been reported in *Clock*^*-/-*^ mice [10], but this might reflect SCN-driven oscillations, whereas our *in vitro* results establish that *Clock*^*-/-*^ peripheral tissues are capable of truly autonomous circadian oscillation. A further specific implication of our results, consistent with previous work but at times underappreciated, is that *Clock*^*-/-*^ mice are not appropriate for experimental studies aiming to eliminate peripheral circadian clock function, because peripheral cells from these mice are in fact rhythmic; instead, such studies should use *Bmal1*^*-/-*^ or *Cry1*^*-/-*^;*Cry2*^*-/-*^ mice, as lack of rhythmicity has been demonstrated in peripheral cells from these mice [16,18]. Finally, our *in vivo* results demonstrate tissue-specific differences in the role of NPAS2 in the circadian clock, and further studies are needed to elucidate these tissue-specific mechanisms.

## Materials and Methods

### Ethics statement

Mouse studies were approved by the Institutional Animal Care and Use Committee at University of California, San Diego (Protocol number: S07365). Every effort was made to minimize the number of animals used, and their suffering.

### Animals

*Clock*^*-/-*^ mice [10] were crossed to mPer2^Luc^ -SV40 reporter mice [19,23]. From the heterozygous offspring, we created a double homozygous *Clock*^*-/-*^; *mPer2*^*Luc*^ and a *Clock*^*+/+*^; *mPer2*^*Luc*^ line, both with the same mixed genetic background. Henceforth, for convenience, these mice will be referred to as *Clock*^*-/-*^ and WT. All experiments for this study were carried out in 2–4 month old male mice, except SCN cultures which were from neonatal (3–6 day old) male and female mice. Mice were maintained in LD 12:12 cycles (12 h light, 12 h dark, lights on at 06:00 hr) with *ad libitum* access to food and water.

### Primary fibroblast culture

Primary fibroblasts were obtained from tail or ears. Tissues from each mouse were chopped into small pieces with a scalpel and incubated twice in a 0.25% trypsin solution for 30 min at 37°C. The tryspin digestion was stopped by adding culture medium (high glucose, pyruvate DMEM [Gibco, #11995] with 5% FBS and 50 U/ml penicillin, 50 μg/ml streptomycin). Cells were centrifuged (0.5 x g, 5 min, room temperature) and resuspended in fresh culture medium. About 2x10^5^ cells/dish were seeded onto 35 mm dishes and incubated at 37°C with 5% CO_2_. When cells reached ~70% confluence, culture medium was replaced with explant medium formulated for equilibration with 5% CO_2_ (high glucose DMEM [Mediatech, Manassas, VA, USA], 14 mM sodium carbonate, 10 mM HEPES, 52 U/ml penicillin, 52 μg/ml streptomycin, 4 mM L-glutamine, 2% B-27 [GIBCO, Grand Island, NY, USA], 0.1 mM luciferin [BioSynth, Itasca, IL, USA]).

### Tissue culture

Tissues were isolated and kept in half-frozen Hank’s Balanced Salt Solution (HBSS). 300 μm brain slices were prepared with a vibratome (Leica VT1200S, Buffalo Grove, IL, USA). Tissues were immediately transferred to tissue culture inserts (EMD Millipore, Billerica, MA, USA) and cultured in 35 mm dishes containing 1 ml of explant medium formulated for equilibration with air (high glucose DMEM [Mediatech, Manassas, VA, USA], 4 mM sodium carbonate, 10 mM HEPES, 52 U/ml penicillin, 52 μg/ml streptomycin, 4 mM L-glutamine, 2% B-27 [GIBCO, Grand Island, NY, USA], 0.1 mM luciferin [BioSynth, Itasca, IL, USA]). When indicated, tissues were treated with 10 μM forskolin for 2 hours in order to enhance rhythmicity.

### mPer2^Luc^ measurements in LumiCycle luminometer

Luminescence measurements were taken at 10 min intervals using a LumiCycle luminometer (Actimetrics) that was placed inside a 37°C incubator without CO_2_. Period, peak phases, goodness of fit, and amplitude were determined over 7 days by fitting a sine wave [Sin fit (Damped) for period, phase, and damping constant (days to reach 1/e of initial amplitude), or LM fit (Sin) for amplitude] to 24 h running average baseline-subtracted data using LumiCycle Analysis software (Actimetrics, Wilmette, IL, USA). The first day of measurement was excluded from analyses. Amplitude was normalized to total brightness in order to account for different sizes of brain tissue and technical differences between slices. Explants failing to show significant χ^2^ periodogram values near 24 h [24], a goodness of fit >0, or a minimum of two mPer2^Luc^ peaks were determined to be arrhythmic and were excluded from further quantification.

### mPer2^Luc^ bioluminescence imaging

Single-cell mPer2^Luc^ measurements were carried out as described elsewhere [25,26]. Briefly, a sealed culture in air-equilibrated explant medium was placed on the stage of an inverted microscope (Olympus IX-71, Tokyo, Japan) in a dark, windowless room. A heated lucite chamber, custom-engineered to fit around the microscope stage (Solent Scientific, Segensworth, UK), kept the sample at a constant 36°C. Light from the sample was collected by an Olympus 4x XLFLUOR objective (NA 0.28) and transmitted directly to a cooled charge-coupled-device (CCD) camera (Spectral Instruments, Tucson, AZ, USA) mounted on the bottom port of the microscope. The camera contained a back-thinned CCD thermoelectrically cooled to -90°C with a rated quantum efficiency of 92% at 560 nm. The signal-to-noise ratio was increased by 4 × 4 binning of the 1056 × 1032 pixel array. Images were collected at intervals of 30 min, with 29.5 min exposure duration, for 4–7 days. Images were acquired and saved to a computer with SI Image SGL D software (Spectral Instruments), and analyzed with MetaMorph (Molecular Devices, Sunnyvale, CA, USA). Period, goodness of fit, and amplitude were determined over 7 days by fitting a sine wave [Sin fit (Damped) for period, phase, and damping constant (time to reach 1/e of initial amplitude), or LM fit (Sin) for amplitude] to 24 h running average baseline-subtracted data using LumiCycle Analysis software (Actimetrics, Wilmette, IL, USA). Cells failing to show significant χ^2^ periodogram values near 24 h [24], a goodness of fit >0, or a minimum of two mPer2^Luc^ peaks were determined to be arrhythmic and were excluded from further quantification.

### Quantitative real-time PCR

Quantitative real-time PCR (qPCR) was performed with a CFX384 thermocycler system (Bio-Rad, Hercules, CA) with GoTaq SYBR Master Mix (Promega, Madison, WI). Relative quantification of expression levels by a modified ΔΔCT calculation was performed as described [27]. *ß-Actin* was used as a reference gene. PCR primer sequences are listed in S2 Table.

### NPAS2 knockdown with lentiviral shRNA

Primary fibroblasts were transfected with either *Npas2* shRNA (GCTCCGAGAATCGAATGTGAT and GCAAGAACATTCCGAAGTTTA) or scrambled control shRNA (TCGTTTACCACCTCCTGCA) lentiviral particles that also contained a GFP marker sequence. Cells were split and grown until ~70% confluency was reached (max. 48 hours). Medium was reduced and polybrene (4 μg/ml) was added. 10 μl of each *Npas2* and 20 μl of the control virus stock solution were added and cells were incubated for 3 hours at 37°C. Afterwards medium was changed. Seven days after transfection, cells were used for single cell mPer2^Luc^ measurements. The efficiency of the *Npas2* knockdown was tested with qPCR after cells were sorted by flow cytometry according to their expression of GFP (S5 Fig).

### Data analysis

Statistical analyses were conducted using GraphPad Prism. Rhythmicity and phase of qPCR clock gene expression profiles were determined using CircWave v1.4 (developed by Roelof Hut, University of Groningen, Netherlands, http://www.euclock.org/results/item/circ-wave.html). CircWave v1.4 fits data to a sine wave with added harmonics. Significance of the curve fit is tested against a fitted horizontal line through the overall average. Phase is calculated as the Center of Gravity of the fitted wave form. For raster plots, bioluminescence intensity values were normalized to mean intensity for each cell. Using Gene Cluster 3.0 and Treeview (developed by Dr. Michael Eisen while at Stanford University, USA), data were color coded, with green for positive and red for negative values. Details about statistical tests used for individual experiments are indicated in the figure legends.

## Supporting Information

S1 Fig Raw data of the two representative mPer2^Luc^ bioluminescence rhythms of rhythmic SCN cells in slices (A), dispersed SCN cells (B), and fibroblasts (C) shown in Figs 1B, 2B and 3B. (TIF)

S2 Fig Raw data of the two representative mPer2^Luc^ bioluminescence rhythms of rhythmic organotypic liver (black), lung (blue), kidney (red), and adrenal (green) slice cultures from wild type (left) and *Clock*^*-/-*^ (right) mice shown in Fig 4A. After ~7 culture days, samples were treated with 10 μM forskolin.
(TIF)

S3 Fig Circadian mPer2^Luc^ rhythm period, amplitude, damping constant (days to reach 1/e of initial amplitude), and phase of first peak after forskolin treatment, and % of slices from wild type (unfilled bars) and *Clock*^*-/-*^ mice (filled bars) that were significantly rhythmic before forskolin treatment (culture days 1–7). Data are shown as mean ± SEM; *p≤0.05, **p≤0.01, ***p≤0.001 (student’s t-test); or % of cells rhythmic; n = 8.
(TIF)

S4 Fig Peripheral organs of *Clock*^*-/-*^ mice can exhibit circadian rhythms of clock gene and clock controlled gene expression *in vivo*. Over the course of one day, livers, lungs, kidneys, and adrenals of wild type (filled symbols) and *Clock*^*-/-*^ (unfilled symbols) mice were collected every 4 hours starting at ZT1. mRNA levels of *Npas2* (blue), *Bmal1* (green), *Per2* (red) and various clock controlled genes (black) were measured by qPCR. Y-axis scales are adjusted to amplitudes for better visualization of data. Data are shown as mean ± SEM and are superimposed with sine wave fits (solid lines: wild type, dashed lines: *Clock*^*-/-*^), n = 3 per time point. Gray shading represents dark phase.
(TIF)

S5 Fig Lentiviral vectors expressing shRNAs stably suppress *Npas2* expression in mouse fibroblasts. The average efficiency of the *Npas2*-knockdown was 60%. The efficiency of the *Npas2* knockdown was tested with qPCR after cells were sorted by flow cytometry according to their expression of GFP. Data are shown as mean ± SEM; *p≤0.05 (student’s t-test); n = 4.
(TIF)

S1 Table Mean of gene expression, phase of gene expression rhythms, and rhythmicity *p*-values for mRNA expression data shown in Fig 4. Values are based on sine wave fits generated with CircWave v1.4 software. Highlighted in bold are significant differences (*p* ≤0.05) between wild-type and *Clock*^*-/-*^ means (student’s t-test, degrees of freedom: 17; analyzed with GraphPad Prism), and significant rhythmicity (*p* ≤0.05) (analyzed with CircWave v.14). Data are presented as mean ± SD, 3 animals per time point: n = 18.
(DOCX)

S2 Table qPCR primer sequences. (DOCX)

S1 Dataset Bioluminescence raw data–SCN cells explants from wild type, *Clock*^*-/-*^, and *Bmal1*^*-/-*^ mice. (XLSX)

S2 Dataset Bioluminescence raw data–Dispersed SCN cells from wild type, *Clock*^*-/-*^, and *Bmal1*^*-/-*^ mice. (XLSX)

S3 Dataset Bioluminescence raw data–Dispersed fibroblasts from wild type and *Clock*^*-/-*^ mice. (XLSX)

S4 Dataset Bioluminescence raw data–Cultured tissues from wild type and *Clock*^*-/-*^ mice. (XLSX)

S5 Dataset Bioluminescence raw data–Dispersed fibroblasts–Npas2-knockdown. (XLSX)
