# Supplementary figures and images for: NPAS2 Compensates for Loss of CLOCK in Peripheral Circadian Oscillators
Source: PLoS Genet. 2016 Feb 19;12(2):e1005882. doi: 10.1371/journal.pgen.1005882 (PMC4760943; doi:10.1371/journal.pgen.1005882)

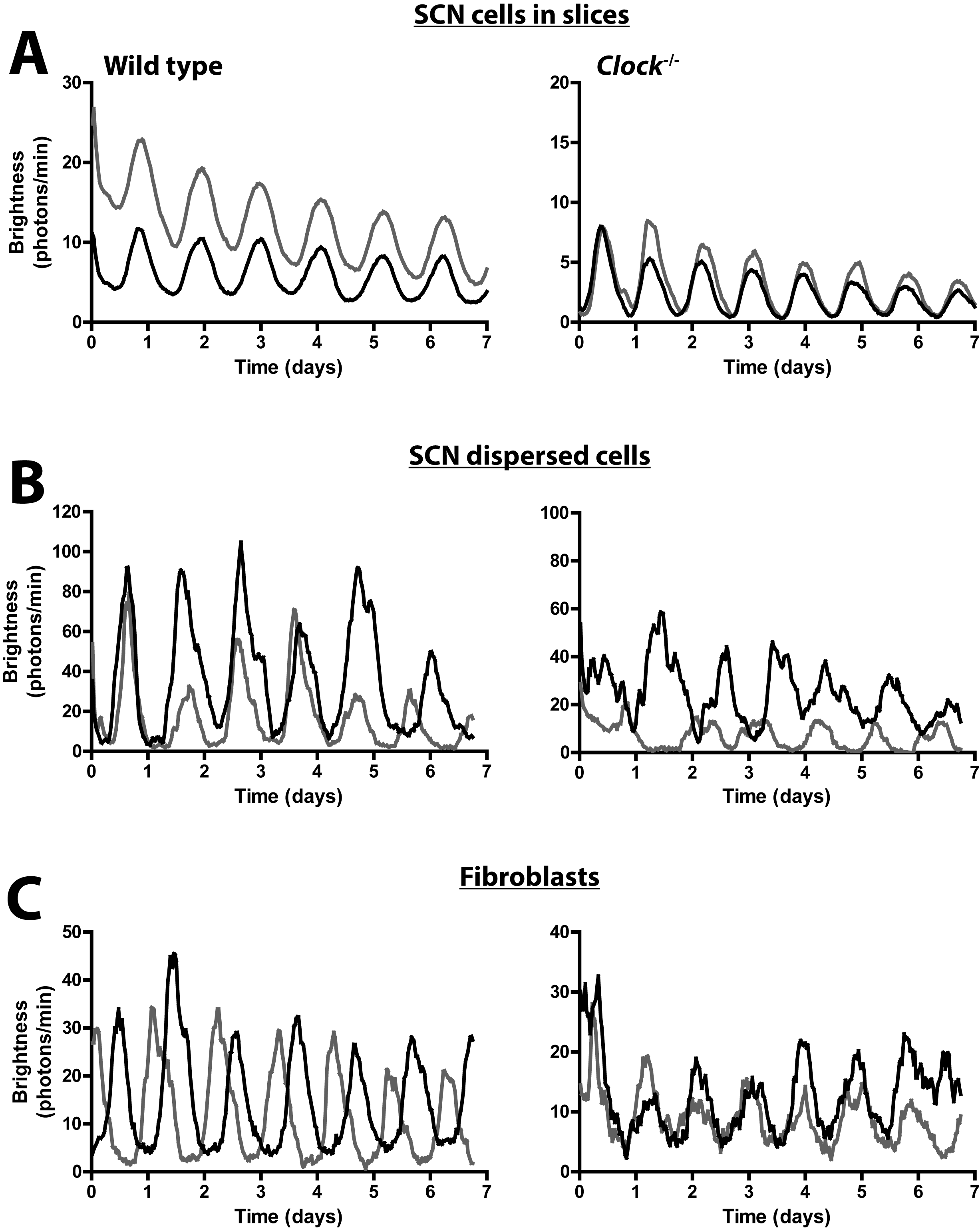

Supplement: S1 Fig — (TIF) [file pgen.1005882.s001.tif]

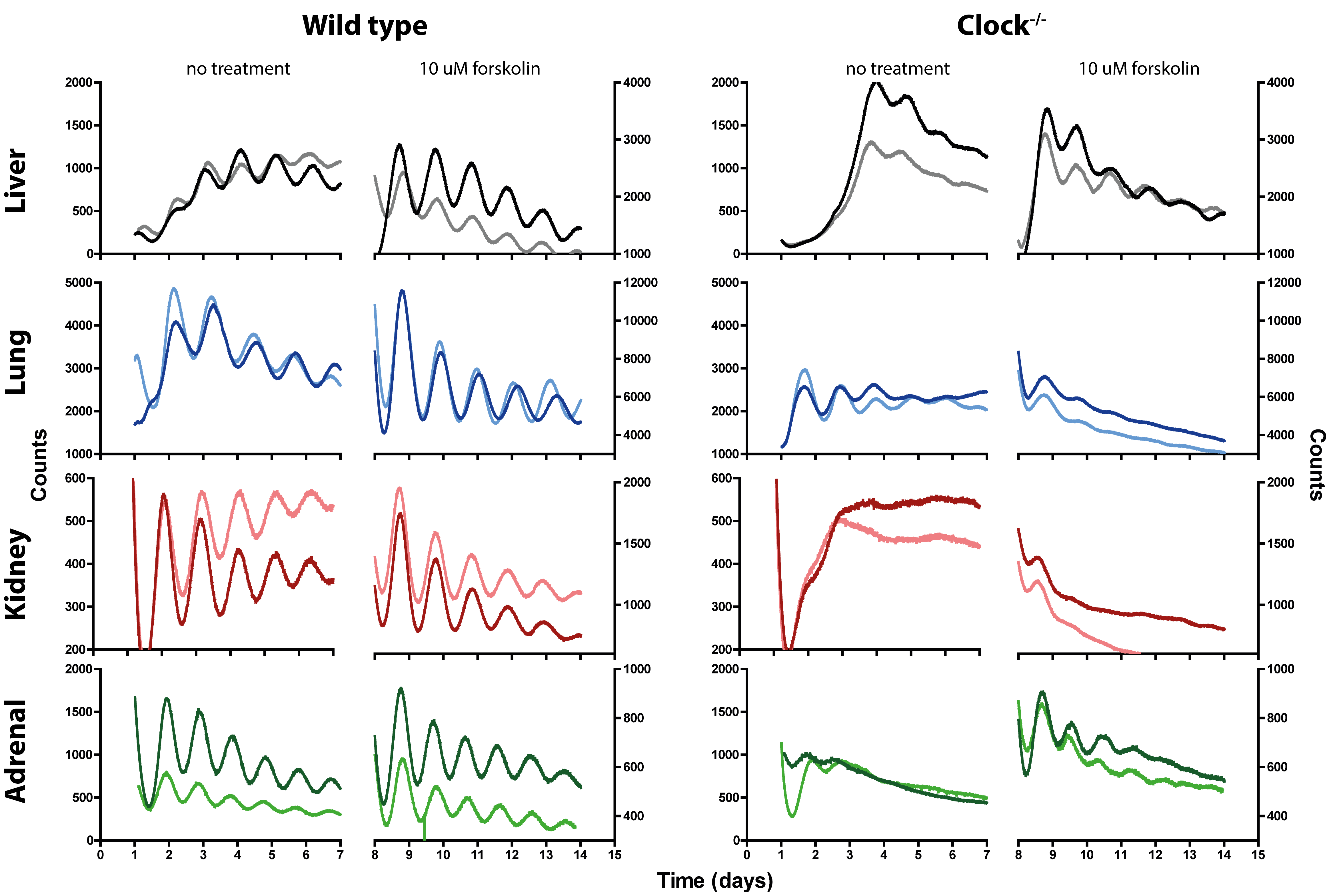

Supplement: S2 Fig — After ~7 culture days, samples were treated with 10 μM forskolin. (TIF) [file pgen.1005882.s002.tif]

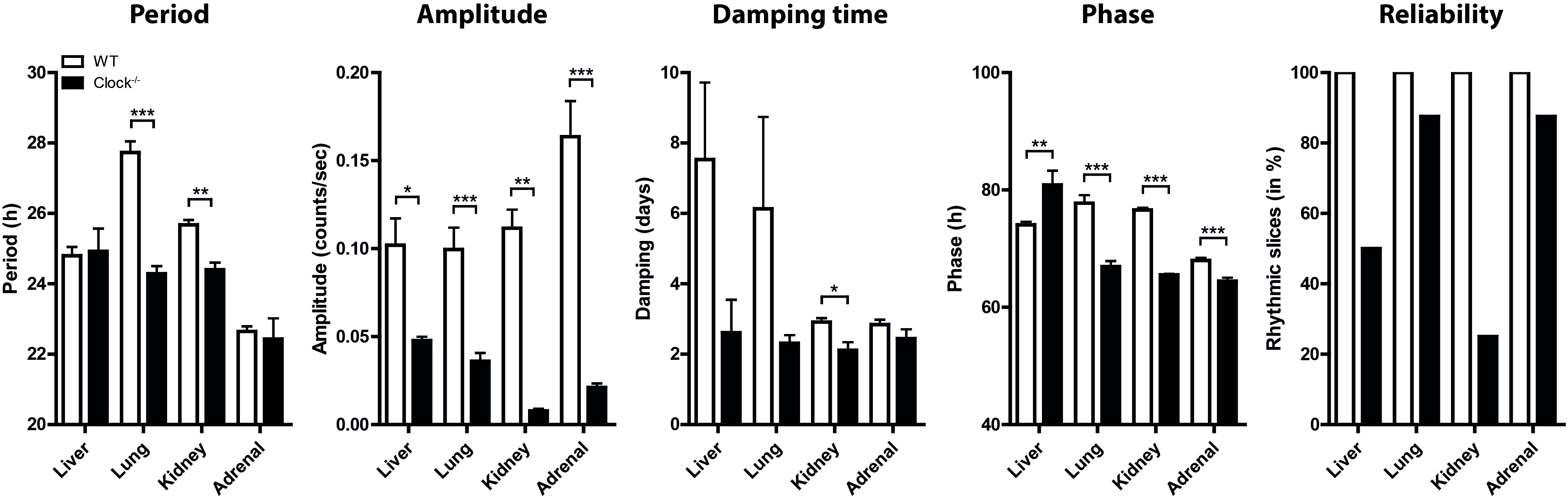

Supplement: S3 Fig — Data are shown as mean ± SEM; *p≤0.05, **p≤0.01, ***p≤0.001 (student’s t-test); or % of cells rhythmic; n = 8. (TIF) [file pgen.1005882.s003.tif]

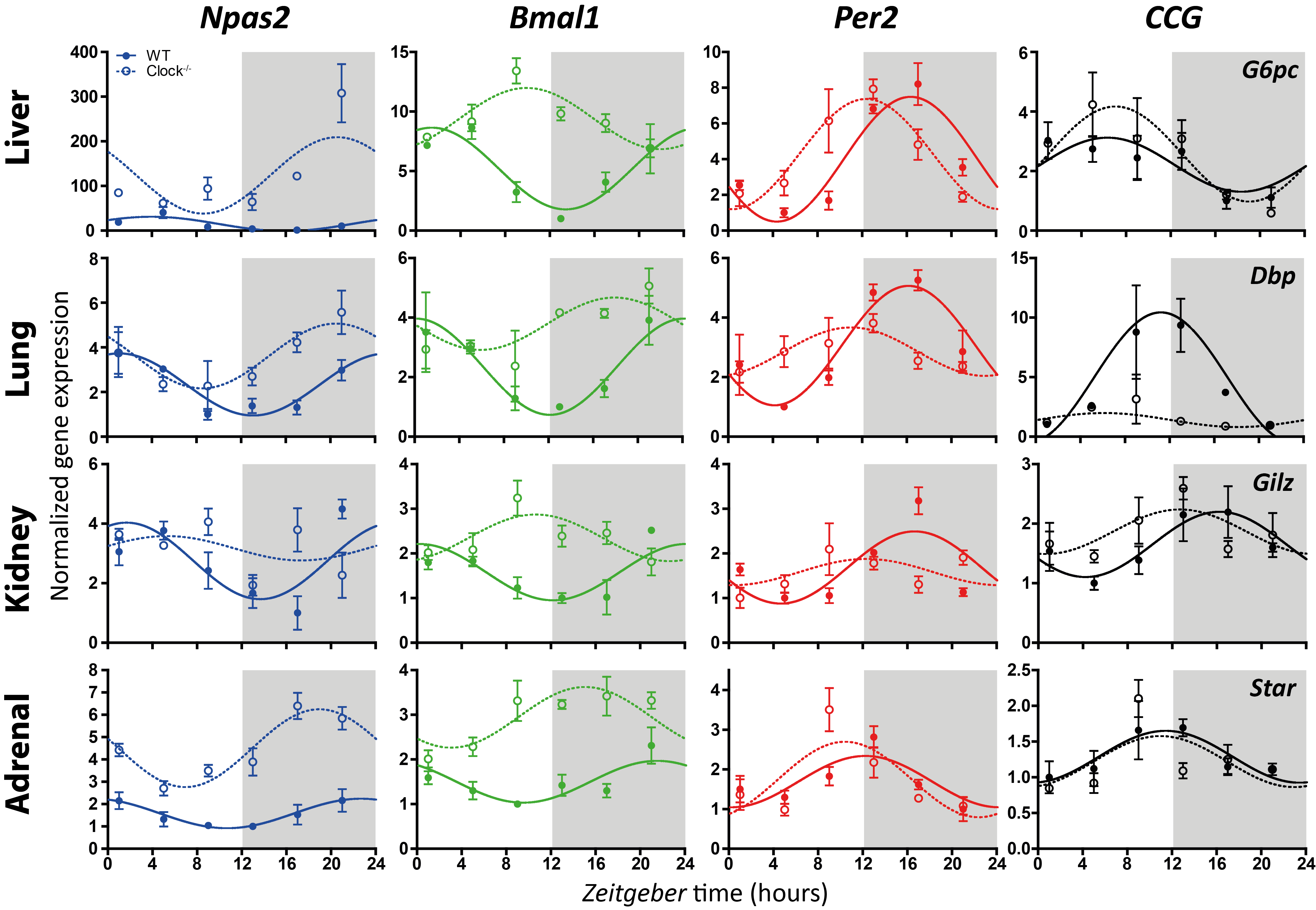

Supplement: S4 Fig — Over the course of one day, livers, lungs, kidneys, and adrenals of wild type (filled symbols) and Clock-/- (unfilled symbols) mice were collected every 4 hours starting at ZT1. mRNA levels of Npas2 (blue), Bmal1 (green), Per2 (red) and various clock controlled genes (black) were measured by qPCR. Y-axis scales are adjusted to amplitudes for better visualization of data. Data are shown as mean ± SEM and are superimposed with sine wave fits (solid lines: wild type, dashed lines: Clock-/-), n = 3 per time point. Gray shading represents dark phase. (TIF) [file pgen.1005882.s004.tif]

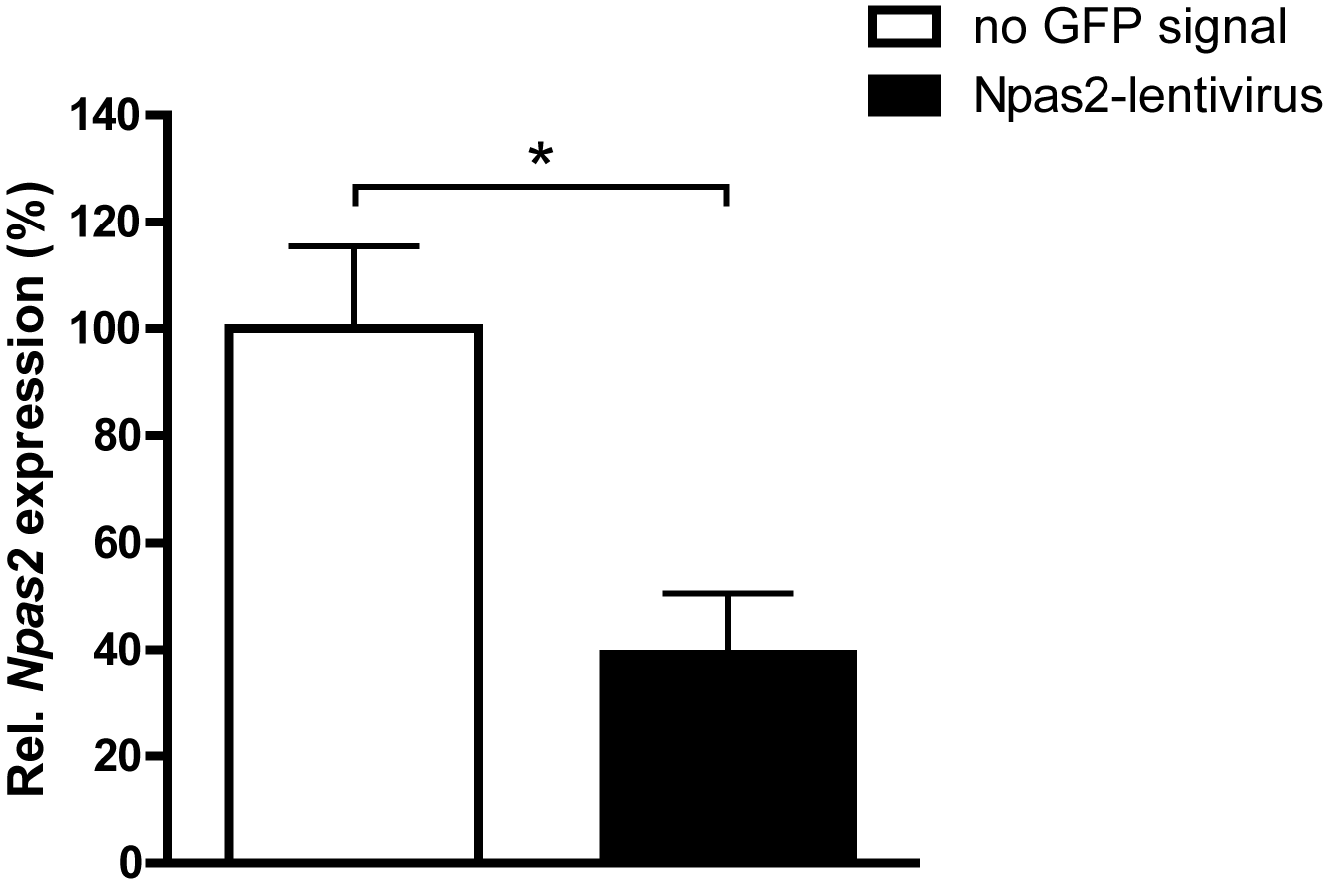

Supplement: S5 Fig — The average efficiency of the Npas2-knockdown was 60%. The efficiency of the Npas2 knockdown was tested with qPCR after cells were sorted by flow cytometry according to their expression of GFP. Data are shown as mean ± SEM; *p≤0.05 (student’s t-test); n = 4. (TIF) [file pgen.1005882.s005.tif]
