# Supplementary material for: NPAS2 Compensates for Loss of CLOCK in Peripheral Circadian Oscillators
Source: PLoS Genet. 2016 Feb 19;12(2):e1005882. doi: 10.1371/journal.pgen.1005882 (PMC4760943; doi:10.1371/journal.pgen.1005882)
Supplement: S1 Table — Values are based on sine wave fits generated with CircWave v1.4 software. Highlighted in bold are significant differences (p ≤0.05) between wild-type and Clock-/- means (student’s t-test, degrees of freedom: 17; analyzed with GraphPad Prism), and significant rhythmicity (p ≤0.05) (analyzed with CircWave v.14). Data are presented as mean ± SD, 3 animals per time point: n = 18. (DOCX) [file pgen.1005882.s006.docx]

| **LIVER** |  |  |  |  |  |  |  |
| --- | --- | --- | --- | --- | --- | --- | --- |
| ***Npas2*** | | | | ***Bmal1*** | | | |
|  | Data Mean | Phase  (ZT) | Rhythmicity *p*-value |  | Data Mean | Phase  (ZT) | Rhythmicity *p*-value |
| WT | 13.7 ± 16.1 | 3.8 | **0.00374** | WT | 5.2 ± 3.1 | 1.3 | **0.000262** |
| Clock-KO | **122.4 ± 97.8** | 20.5 | **0.020487** | Clock-KO | **9.4 ± 2.5** | 9.8 | **0.001649** |
| ***Per2*** | | | | ***G6pc*** | | | |
|  | Data Mean | Phase  (ZT) | Rhythmicity *p*-value |  | Data Mean | Phase  (ZT) | Rhythmicity *p*-value |
| WT | 4.0 ± 2.9 | 16.2 | **9.00E-06** | WT | 2.2 ± 1.1 | 6.2 | **0.043968** |
| Clock-KO | 4.3 ± 2.7 | 12.2 | **0.000121** | Clock-KO | 2.5 ± 1.9 | 6.9 | **0.00787** |

| **LUNG** |  |  |  |  |  | |  |  |
| --- | --- | --- | --- | --- | --- | --- | --- | --- |
| ***Npas2*** | | | | ***Bmal1*** | | | | |
|  | Data Mean | Phase  (ZT) | Rhythmicity *p*-value |  | Data Mean | Phase  (ZT) | | Rhythmicity *p*-value |
| WT | 2.3 ± 1.4 | 0.6 | **0.002291** | WT | 2.4 ± 1.6 | 23.7 | | **0.003098** |
| Clock-KO | **3.6 ± 1.5** | 20.5 | **0.017246** | Clock-KO | **3.8 ± 1.1** | 18.6 | | **0.040169** |
| ***Per2*** | | | | ***Dbp*** | | | | |
|  | Data Mean | Phase | Rhythmicity *p*-value |  | Data Mean | Phase | | Rhythmicity *p*-value |
| WT | 3.1 ± 1.8 | 16.5 | **0.000224** | WT | 4.6 ± 4.5 | 11.4 | | **3.50E-05** |
| Clock-KO | 2.8 ± 0.9 | 12.4 | **0.023953** | Clock-KO | **1.3 ± 0.6** | 4.7 | | **0.004414** |

| **KIDNEY** |  |  |  |  | |  |  |  |  |
| --- | --- | --- | --- | --- | --- | --- | --- | --- | --- |
| ***Npas2*** | | | | ***Bmal1*** | | | | |  |
|  | Data Mean | Phase  (ZT) | Rhythmicity *p*-value |  | | Data Mean | Phase  (ZT) | Rhythmicity *p*-value |  |
| WT | 2.8 ± 1.4 | 1.6 | **0.0129399** | WT | | 1.6 ± 0.6 | 0.2 | **0.003889** |  |
| Clock-KO | 3.2 ± 1.1 | n.a. | 0.555993 | Clock-KO | | **2.3 ± 0.6** | 10.6 | **0.039375** |  |
| ***Per2*** | | | | | ***Gilz*** | | | | |
|  | Data Mean | Phase  (ZT) | Rhythmicity *p*-value |  | | Data Mean | Phase  (ZT) | Rhythmicity *p*-value |  |
| WT | 1.7 ± 0.8 | 16.6 | **0.004794** | WT | | 1.6 ± 0.6 | 16.2 | **0.018514** |  |
| Clock-KO | 1.6 ± 0.6 | n.a. | 0.335424 | Clock-KO | | 1.9 ± 0.6 | n.a. | 0.140443 |  |

| **ADRENAL** |  | |  |  |  |  |  |  |
| --- | --- | --- | --- | --- | --- | --- | --- | --- |
| ***Npas2*** | | | | | ***Bmal1*** | | | |
|  | Data Mean | | Phase  (ZT) | Rhythmicity *p*-value |  | Data Mean | Phase  (ZT) | Rhythmicity *p*-value |
| WT | 1.5 ± 0.7 | | 22.6 | **0.00867** | WT | 1.5 ± 0.5 | 21.4 | **0.019697** |
| Clock-KO | **4.5 ± 1.5** | | 18.90 | **5.10E-05** | Clock-KO | **2.9 ± 0.7** | 14.9 | **0.008193** |
| ***Per2*** | | | | | ***Star*** | | | |
|  | | Data Mean | Phase  (ZT) | Rhythmicity *p*-value |  | Data Mean | Phase  (ZT) | Rhythmicity *p*-value |
| WT | | 1.7 ± 0.7 | 12.1 | **0.009368** | WT | 1.3 ± 0.4 | 11.3 | **0.033384** |
| Clock-KO | | 1.7 ± 1.0 | 10.3 | **0.011083** | Clock-KO | 1.2 ± 0.5 | n.a. | 0.07896 |
