# Supplementary material for: NPAS2 Compensates for Loss of CLOCK in Peripheral Circadian Oscillators
Source: PLoS Genet. 2016 Feb 19;12(2):e1005882. doi: 10.1371/journal.pgen.1005882 (PMC4760943; doi:10.1371/journal.pgen.1005882)
Supplement: S2 Table — (DOCX) [file pgen.1005882.s007.docx]

| **Gene** | **Forward primer** | **Reverse primer** |
| --- | --- | --- |
| *Npas2* | CAGGACTGGAAGCCATCATT | GATGCTGCCGTCTGTTGTC |
| *Bmal1* | CCTAATTCTCAGGGCAGCAGAT | TCCAGTCTTGGCATCAATGAGT |
| *Per2* | GCCAAGTTTGTGGAGTTCCTG | CTTGCACCTTGACCAGGTAGG |
| *G6pc* | CGACTCGCTATCTCCAAGTGA | GTTGAACCAGTCTCCGACCA |
| *Dbp* | AATGACCTTTGAACCTGATCCCGCT | GCTCCAGTACTTCTCATCCTTCTGT |
| *Gilz* | AGCAGGCCATGGACCTCGTGA | CGCGCTCCAGCTGGGAGTTC |
| *Star* | TATCTCTGCCGTCTGGGATGT | GCCATCTTGGGCTACAGAATG |
| *β-actin* | CCCTGAAGTACCCCATTGAA | AGGTGTGGTGCCAGATCTTC |
